# Integrity of the Human Faecal Microbiota following Long-Term Sample Storage

**DOI:** 10.1371/journal.pone.0163666

**Published:** 2016-10-04

**Authors:** Elahe Kia, Brett Wagner Mackenzie, Danielle Middleton, Anna Lau, David W. Waite, Gillian Lewis, Yih-Kai Chan, Marta Silvestre, Garth J. S. Cooper, Sally D. Poppitt, Michael W. Taylor

**Affiliations:** 1 School of Biological Sciences, University of Auckland, Auckland, New Zealand; 2 Department of Surgery, University of Auckland, Auckland, New Zealand; 3 Human Nutrition Unit, University of Auckland, Auckland, New Zealand; Universidad Andres Bello, CHILE

## Abstract

In studies of the human microbiome, faecal samples are frequently used as a non-invasive proxy for the study of the intestinal microbiota. To obtain reliable insights, the need for bacterial DNA of high quality and integrity following appropriate faecal sample collection and preservation steps is paramount. In a study of dietary mineral balance in the context of type 2 diabetes (T2D), faecal samples were collected from healthy and T2D individuals throughout a 13-day residential trial. These samples were freeze-dried, then stored mostly at -20°C from the trial date in 2000/2001 until the current research in 2014. Given the relative antiquity of these samples (~14 years), we sought to evaluate DNA quality and comparability to freshly collected human faecal samples. Following the extraction of bacterial DNA, gel electrophoresis indicated that our DNA extracts were more sheared than extracts made from freshly collected faecal samples, but still of sufficiently high molecular weight to support amplicon-based studies. Likewise, spectrophotometric assessment of extracts revealed that they were of high quality and quantity. A subset of bacterial 16S rRNA gene amplicons were sequenced using Illumina MiSeq and compared against publicly available sequence data representing a similar cohort analysed by the American Gut Project (AGP). Notably, our bacterial community profiles were highly consistent with those from the AGP data. Our results suggest that when faecal specimens are stored appropriately, the microbial profiles are preserved and robust to extended storage periods.

## Introduction

Our understanding of the gut microbiota and its role in human health has benefitted immensely from recent technological advances. The advent of next-generation sequencing, in particular, has revolutionised this field [1]. However, the results of sequencing studies are only meaningful if the preceding steps, starting with sample collection and preservation, yield samples of high integrity. Faecal samples, commonly used as a non-invasive proxy for the study of the intestinal microbiota, are routinely frozen following collection, yet even this may affect the recorded ratios of key bacterial taxa [2]. Other facets of the experimental protocol, such as DNA preservation and extraction methods, choice of PCR primers, and sequencing platform also have the potential to influence results [3–6].

In a study of complete dietary macronutrient and trace mineral balance in the context of type 2 diabetes (T2D), blood, urine and faecal samples were collected from individuals throughout a 13-day residential trial [7]. After collection, faeces were immediately frozen at -20°C, then subsequently freeze-dried using a commercial service. Although analysis of the microbiota was not an initial objective of this study, samples were collected in accordance with standard microbiological practice and remained mostly at -20°C from the trial date in 2000/2001 until the current research in 2014. Samples were thus ~13–14 years old at the time of microbiota analysis. In total, 454 faecal samples were analysed from the 40 males participating in the study (20 individuals with T2D plus 20 overweight but otherwise healthy individuals) (S1 Table). Full details of the study cohort were reported previously [7].

Given the relative antiquity of the faecal samples, we sought to determine whether they would yield reliable data on bacterial community composition. Here, we describe our systematic evaluation of sample integrity, using a three-pronged approach. Firstly, extracted DNA was visualised following gel electrophoresis to assess the extent of shearing. Next, DNA quantity and quality were assessed spectrophotometrically. The final, and arguably most important step, was to compare our obtained 16S rRNA gene amplicon data with publicly available sequence data from a study cohort with similar demographic and health characteristics. We conclude that, despite storage of these freeze-dried samples for more than a decade, our sequence data are entirely reliable and analysis of the full bank of samples—which will yield crucial insights into the relationship between T2D, diet and the microbiota—is warranted.

## Materials and Methods

### Molecular analyses

For evaluation of sample integrity, DNA was extracted from 454 freeze-dried faecal samples derived from Cooper *et al*. [7]. Ethics approval was granted in 2001 by the New Zealand Ministry of Health Northern X Committee (Approval number 2001/026). Written informed consent was obtained from the healthy and T2D cohorts for their samples to be used in this study. DNA was extracted from 50 mg of each faecal sample using the MoBio PowerSoil^®^ DNA Isolation Kit. DNA integrity was visualised by electrophoresing 4 μL of extracted DNA at 100 V for 60 min on a 1% agarose gel (w/v) stained with SYBR SAFE. Quantification of DNA was achieved spectrophotometrically using the Nanodrop^®^ ND-1000 (Nanodrop Technologies Inc., Wilmington, USA), which also provided a 260/280 nm absorbance ratio for assessment of DNA purity.

### Comparison of 16S rRNA gene sequences with data obtained from a similar cohort

We selected 13 of the freeze-dried faecal samples derived from the cohort of healthy individuals of Cooper *et al*. [7] (S1 Table) and sequenced the 16S rRNA genes. These represented all of the samples provided by healthy individuals on Day 1 (baseline) of the study, and were collected prior to treatment or potential influence of dietary intervention. PCR primers 341F and 806R were used to amplify the V3-V4 region of 16S rRNA genes due to their excellent phylogenetic breadth [8]. PCR amplicons were purified using AMPure magnetic beads (Agencourt) and sequenced *via* Illumina MiSeq 2x300 bp paired-end sequencing; sequencing was carried out by the Centre for Genomics, Proteomics and Metabolomics through NZ Genomics Ltd at the University of Auckland. Sequence data were deposited in the NCBI Sequence Read Archive (BioProject ID PRJNA321230).

In the absence of an appropriate microbiota data set for New Zealand individuals, we compared our sequence data with those obtained from faecal samples analysed by the American Gut Project (AGP). A current summary of the AGP is available at https://www.microbio.me/AmericanGut/static/img/mod1_main.pdf. In addition to the extensive metadata collected by the AGP which allowed us to stratify our cohort as rigidly as possible, the post-storage processing of faecal samples from the AGP is similar to the approach used in our study, i.e. the same DNA extraction kit and Illumina sequencing technologies were employed.

We stratified the AGP cohort according to the inclusion criteria outlined in Cooper *et al*. [7] for healthy (non-T2D) individuals. Briefly, faecal samples were included from male participants aged between 30 and 68 years; body mass index (BMI) between 21.0–42.5 kg/m^2^; no participants were morbidly obese (BMI ≥45 kg/m^2^); no history of diabetes (type 1 or 2), significant cardiac, hepatic, gastrointestinal, haematological, respiratory, endocrine, or psychiatric disease, as well as autoimmune disorders or immunosuppressive therapy. Antibiotic history and use was not recorded in the study by Cooper and colleagues, so these were not considered as exclusion criteria for AGP participants. Sequence data from 117 AGP participants were downloaded from the European Nucleotide Archive (S2 Table), and these were used for comparison with data from the 13 overweight but healthy individuals from the earlier study by Cooper and colleagues [7].

The complete 16S rRNA gene data set was analysed according to the AGP and Earth Microbiome Project (EMP) standardised protocols (available at http://nbviewer.jupyter.org/github/biocore/American-Gut/blob/master/ipynb/module2_v1.0.ipynb). Briefly, the forward reads from our data set were trimmed using USEARCH version 7.0 [9] to include only the first 100 bp of the V4 hypervariable region, to be consistent with the AGP sequence data. Primer sequences were removed from both our data and those of the AGP. After preliminary sequence processing of both data sets independently and according to the EMP protocol, closed-reference OTU picking and taxonomic assignment against the Greengenes database (pre-clustered at 97% identity) was carried out using the QIIME command pick_closed_reference_otus.py. Taxonomically assigned biom tables were merged. The Greengenes phylogenetic tree at 97% identity was used in all downstream phylogenetic analyses.

From the original selection of 13 Cooper *et al*. [7] samples and 117 AGP samples, quality filtering during sequence processing resulted in a total of 12 NZ samples (range from 3269 to 18269 sequence reads) and 110 AGP samples (range from 1291 to 131394 reads) for data comparison. All samples were rarefied to 1,290 sequences per sample, and alpha and beta diversity metrics were calculated in QIIME version 1.8 [10]. Additional statistics and visualisation of the data in non-metric multidimensional scaling (MDS) plots were generated using PRIMER version 7.0 [11].

## Results and Discussion

### Shearing of extracted DNA

**Fig 1:**
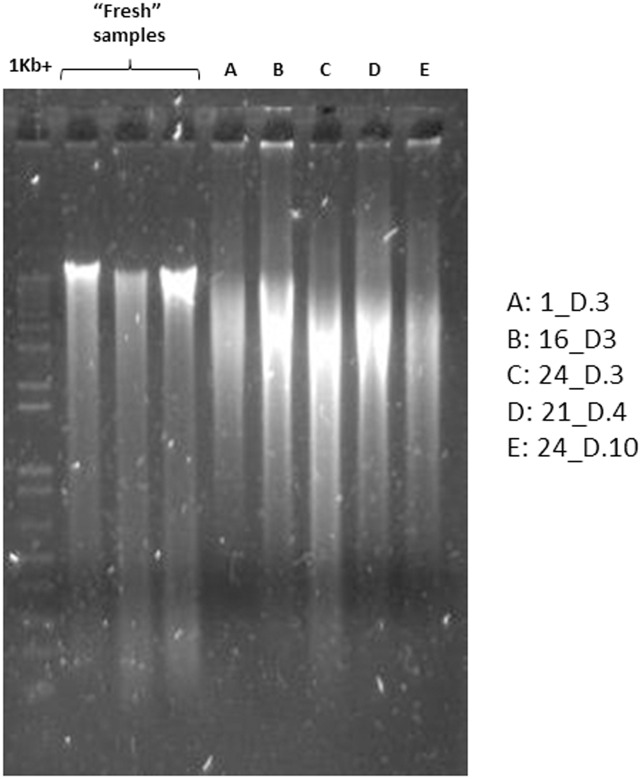
Agarose gel electrophoresis image showing representative DNA extracts obtained from the freeze-dried faecal samples of Cooper *et al*. [7]. DNA extracts from three “fresh” faecal samples are also included for comparison.

Rigorous bead-beating protocols for DNA extraction, such as employed in this study, are used to ensure the lysis of recalcitrant cell types (particularly Gram-positive bacteria). An unwanted side-effect of this harsh treatment can be excessive fragmentation of DNA, visualised as shearing of low molecular weight DNA down an agarose gel following electrophoresis. Highly fragmented DNA can also be a consequence of degraded samples, as routinely encountered in the analysis of ancient DNA [12]. We witnessed some shearing of DNA among the extracts obtained from the freeze-dried faecal samples in this study, which was more than that observed using the same extraction protocol with fresh faecal samples. A representative set of five samples from Cooper *et al*. (2005) [7] is shown in Fig 1, together with extracts of three fresh samples. Variable band strengths can be observed at the top of the gel (reflecting variability in DNA extraction efficiency despite identical faecal input amounts), while even DNA which was sheared was still of higher molecular weight than the 464-bp fragment of the 16S rRNA gene amplified in this study. We therefore deemed it appropriate to proceed with downstream analyses of these DNA extracts, as 16S rRNA gene amplicon analyses do not require the presence of exclusively high molecular weight DNA.

### High quality of extracted DNA

Both quantity and quality of extracted DNA can be easily assessed spectrophotometrically, with a 260/280 nm absorbance ratio of ~1.8 considered to be indicative of pure DNA [13]. The 454 analysed samples yielded a mean 260/280 nm ratio of 1.859 ± 0.003 (mean ± S.E.) (S1 Table). The recorded 260/280 nm absorbance ratios thus indicate the presence of pure DNA, providing further evidence that the integrity of the samples and extracted DNA were sufficient to proceed with subsequent analyses. The concentration (quantity) of extracted DNA was more variable, but overall quite high (79.342 ± 1.4341 ng/μL (mean ± S.E.)) (S1 Table), and certainly more than sufficient for successful PCR amplification.

### Bacterial 16S rRNA gene amplicon profiles are highly consistent with those from another study with a similar cohort

A vital component of our analysis was to determine whether bacterial community profiles were what one might expect for a cohort of this type, with key bacterial taxa present at appropriate abundances. Bacterial 16S rRNA gene amplicon profiles were therefore obtained from DNA extracted out of 12 of the freeze-dried faecal samples (one of the original 13 samples did not return sufficient numbers of sequence reads). These samples—hereafter referred to as “NZ”–represented control (non-T2D) individuals on Day 1 of the diet trial of Cooper *et al*. [7], prior to any potential influence of the dietary intervention.

**Fig 2:**
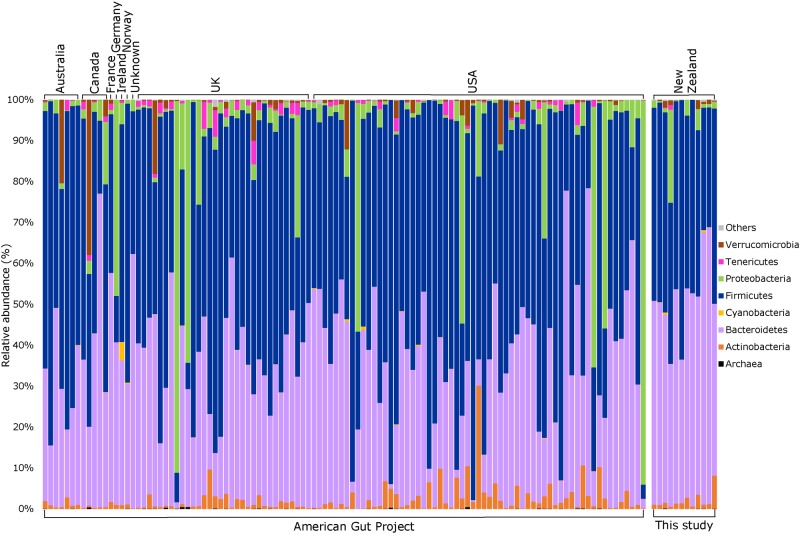
Phylum-level comparison of bacterial community structure between the freeze-dried faecal samples from Cooper *et al*. [7] (labelled “This study”, n = 12) and a matched cohort of individuals from the American Gut Project (n = 110). “Others” represent bacterial phyla which did not comprise >0.1% relative sequence abundance in multiple samples.

**Fig 3:**
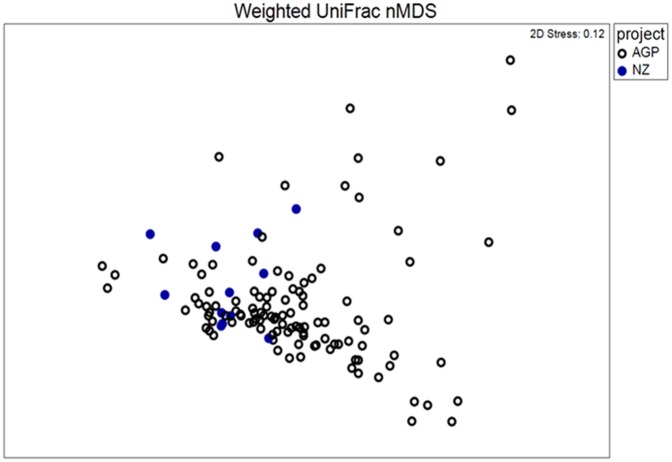
Non-metric multidimensional scaling plot showing overlap in bacterial community structure between the freeze-dried faecal samples from Cooper *et al*. [7] (blue symbols, n = 12) and a matched cohort of individuals from the American Gut Project (open symbols, n = 110). The weighted UniFrac metric was applied.

Consistent with a wealth of published data on the human faecal microbiota [14–16], as well as the American Gut Project (AGP), our bacterial 16S rRNA gene profiles were dominated by members of the phyla *Bacteroidetes* and *Firmicutes*, with members of *Actinobacteria*, *Verrucomicrobia* and *Proteobacteria* present at lower abundances (Fig 2). At phylum level, our data also mirrored those obtained from a cohort of 110 similar individuals (see inclusion criteria in Materials & Methods) within the AGP (Fig 2). Furthermore, visual representation of our taxon-assigned OTU data together with that of the AGP on a non-metric multi-dimensional scaling plot (Fig 3) indicated overlapping microbiota profiles, despite the inherent geographic differences between the two data sets. In addition, an analysis of similarity (ANOSIM) did not find significant differences between the NZ and AGP data sets for either weighted or unweighted UniFrac distances (data not shown); however, a significant difference was obtained when using the Bray-Curtis dissimilarity metric (*p* = 0.003, R = 0.323).

Taken together, these data indicate that preservation of human faecal samples by freeze-drying, followed by frozen storage of these samples for a ~14-year period, can lead to highly reliable descriptions of the bacterial biota. This not only confirms the value of the full Cooper *et al*. [7] sample set for evaluating the contributions of health status and diet to shaping the faecal microbiota, but should also be of potential relevance to other research groups who may question the reliability of faecal samples that they have retained for extended periods of time.

## Supporting Information

S1 Fig Phylum-level comparison of bacterial community structure between the freeze-dried faecal samples from Cooper *et al*. [7] (labelled “NZ”) and the matched cohort of individuals from the American Gut Project, broken down by country of origin. “Others” represent bacterial phyla which did not comprise >0.1% relative sequence abundance in multiple samples.
(PPTX)

S2 Fig Non-metric multidimensional scaling plots showing overlap in bacterial community structure between the freeze-dried faecal samples from Cooper *et al*. [7] (blue symbols, n = 12) and a matched cohort of individuals from the American Gut Project (open symbols, n = 110). Results obtained using three different diversity metrics are shown (weighted UniFrac is the same as Fig 3 in the article).
(PPTX)

S1 Table Details of the 454 samples from Cooper *et al*. [7] alongside respective quantity (ng/uL) and quality (260/280 nm) data of extracted DNA.
(XLSX)

S2 Table Details of samples for 16S rRNA gene sequences obtained in this study and from the American Gut Project. (XLSX)

## References

[1] Kuczynski J, Lauber CL, Walters WA, Parfrey LW, Clemente JC, Gevers D, et al Experimental and analytical tools for studying the human microbiome. Nat Rev Genet. 2012; 13: 47–58. 10.1038/nrg3129 22179717 PMC5119550

[2] Bahl MI, Bergstrom A, Licht TR. Freezing fecal samples prior to DNA extraction affects the *Firmicutes* to *Bacteroidetes* ratio determined by downstream quantitative PCR analysis. FEMS Microbiol Lett. 2012; 329: 193–197. 10.1111/j.1574-6968.2012.02523.x 22325006

[3] Wagner Mackenzie B, Waite DW, Taylor MW. Evaluating variation in human gut microbiota profiles due to DNA extraction method and inter-subject differences. Front Microbiol. 2015; 6: e130 10.3389/fmicb.2015.00130 25741335 PMC4332372

[4] Lozupone CA, Stombaugh J, Gonzalez A, Ackermann G, Wendel D, Vazquez-Baeza Y, et al Meta-analyses of studies of the human microbiota. Genome Res. 2013; 23: 1704–1714. 10.1101/gr.151803.112 23861384 PMC3787266

[5] Werner JJ, Zhou D, Caporaso JG, Knight R, Angenent LT. Comparison of Illumina paired-end and single-direction sequencing for microbial 16S rRNA gene amplicon surveys. ISME J. 2012; 6: 1273–1276. 10.1038/ismej.2011.186 22170427 PMC3379627

[6] Song SJ, Amir A, Metcalf JL, Amato KR. Preservation methods differ in fecal microbiome stability, affecting suitability for field studies. mSystems. 2016; 1: e00021–16. 10.1128/mSystems.00021-16 PMC5069758 27822526

[7] Cooper GJS, Chan YK, Dissanayake AM, Leahy FE, Keogh GF, Frampton CM, et al Demonstration of a hyperglycemia-driven pathogenic abnormality of copper homeostasis in diabetes and its reversibility by selective chelation: Quantitative comparisons between the biology of copper and eight other nutritionally essential elements in normal and diabetic individuals. Diabetes. 2005; 54: 1468–1476. 10.2337/diabetes.54.5.1468 15855335

[8] Klindworth A, Pruesse E, Schweer T, Peplies J, Quast C, Horn M, et al Evaluation of general 16S ribosomal RNA gene PCR primers for classical and next-generation sequencing-based diversity studies. Nucleic Acids Res. 2013; 41: e1 10.1093/nar/gks808 22933715 PMC3592464

[9] Edgar RC. Search and clustering orders of magnitude faster than BLAST. Bioinformatics. 2010; 26: 2460–2461. 10.1093/bioinformatics/btq461 20709691

[10] Caporaso JG, Kuczynski J, Stombaugh J, Bittinger K, Bushman FD, Costello EK, et al QIIME allows analysis of high- throughput community sequencing data Intensity normalization improves color calling in SOLiD sequencing. Nat Methods. 2010; 7: 335–336. 10.1038/nmeth.f.303 20383131 PMC3156573

[11] Clarke K, Gorley R. PRIMER v7: user manual/tutorial. Plymouth: PRIMER-E; 2015 296 p.

[12] Willerslev E, Cooper A. Ancient DNA. Proc R Soc London B. 2005; 272: 3–16. 10.1098/rspb.2004.2813 PMC1634942 15875564

[13] Sambrook J, Russell DW. Molecular Cloning: a laboratory manual, vol. 3. 3rd ed Cold Spring Harbor, New York: Cold Spring Harbor Laboratory Press; 2001.

[14] Lozupone CA, Stombaugh JI, Gordon JI, Jansson JK, Knight R. Diversity, stability and resilience of the human gut microbiota. Nature. 2012; 489: 220–230. 10.1038/nature11550 22972295 PMC3577372

[15] Falony G, Joossens M, Vieira-Silva S, Wang J, Darzi Y, Faust K, et al Population-level analysis of gut microbiome variation. Science. 2016; 352: 560–564. 10.1126/science.aad3503 27126039

[16] Goodrich JK, Waters JL, Poole AC, Sutter JL, Koren O, Blekhman R, et al Human genetics shape the gut microbiome. Cell. 2014; 159: 789–99. 10.1016/j.cell.2014.09.053 25417156 PMC4255478

