# Supplementary material for: Integrity of the Human Faecal Microbiota following Long-Term Sample Storage
Source: PLoS One. 2016 Oct 4;11(10):e0163666. doi: 10.1371/journal.pone.0163666 (PMC5049846; doi:10.1371/journal.pone.0163666)
Supplement: S1 Fig — [7] (labelled “NZ”) and the matched cohort of individuals from the American Gut Project, broken down by country of origin. “Others” represent bacterial phyla which did not comprise >0.1% relative sequence abundance in multiple samples. (PPTX) [file pone.0163666.s001.pptx]

## Slide 1
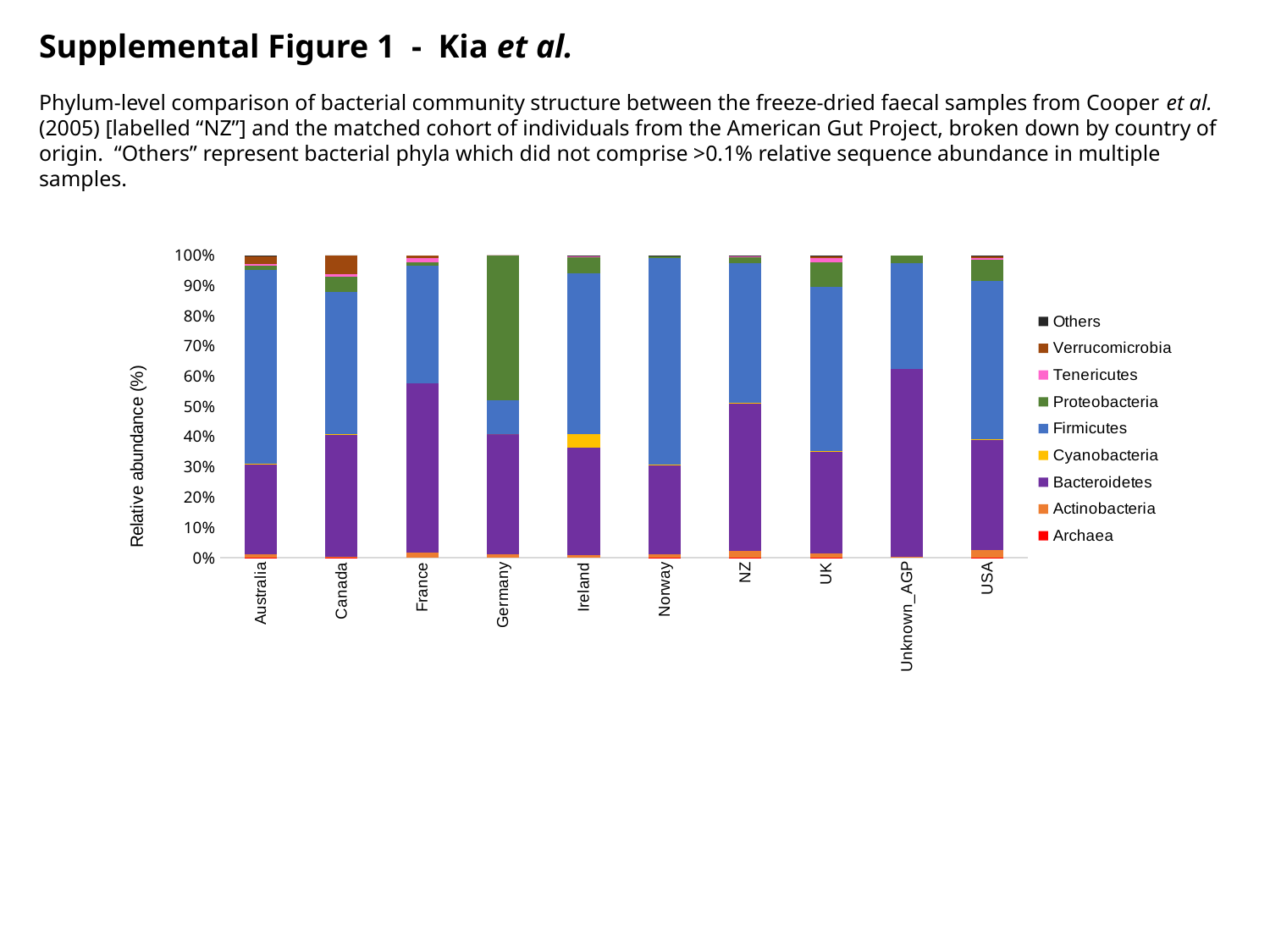

Supplemental Figure 1 - Kia et al.
Phylum-level comparison of bacterial community structure between the freeze-dried faecal samples from Cooper et al. (2005) [labelled “NZ”] and the matched cohort of individuals from the American Gut Project, broken down by country of origin. “Others” represent bacterial phyla which did not comprise >0.1% relative sequence abundance in multiple samples.
### Chart
| Category | Archaea | Actinobacteria | Bacteroidetes | Cyanobacteria | Firmicutes | Proteobacteria | Tenericutes | Verrucomicrobia | Others |
|---|---|---|---|---|---|---|---|---|---|
| Australia | 0.00010626239728 | 0.0123618588835 | 0.297475677718 | 0.000448663455181 | 0.640844904128 | 0.01445168603 | 0.00467554548031 | 0.0294464909795 | 0.0001889109284975 |
| Canada | 0.0004905895995 | 0.0032289715458 | 0.403416287575 | 0.0003211131924 | 0.470635982517 | 0.0515208277585 | 0.00878601373651 | 0.061555615021 | 4.459905450003001e-05 |
| France | 0.0 | 0.0172043010753 | 0.56 | 0.0 | 0.387956989247 | 0.0103225806452 | 0.0154838709677 | 0.00903225806452 | 0.0 |
| Germany | 0.0 | 0.00980199318122 | 0.397849462366 | 8.19564647259e-05 | 0.113198269079 | 0.478920797272 | 1.63912929452e-05 | 3.27825858904e-05 | 9.83477576711e-05 |
| Ireland | 0.0 | 0.00909556751217 | 0.354342813221 | 0.0449654112221 | 0.531898539585 | 0.0527799128875 | 0.00461183704842 | 0.00192159877018 | 0.000384319754035 |
| Norway | 0.000218211971109 | 0.0109324197525 | 0.294542518603 | 0.00325135836952 | 0.682130621686 | 0.00816112771947 | 0.00024003316822 | 0.000196390773998 | 0.00032731795666280007 |
| NZ | 0.00013451859159 | 0.0223188763214 | 0.487809252637 | 0.000751062136379 | 0.462497337653 | 0.0214332955934 | 0.000650173192687 | 0.00332933514186 | 0.0010761487327228 |
| UK | 0.0004158988788642 | 0.0137472157328 | 0.337437930243 | 0.00035421052841 | 0.54451444635 | 0.0807122417547 | 0.0131051262356 | 0.00835445993507 | 0.001358470341169904 |
| Unknown_AGP | 0.0 | 0.0020202020202 | 0.621043771044 | 0.0 | 0.349494949495 | 0.0274410774411 | 0.0 | 0.0 | 0.0 |
| USA | 0.000205431930324 | 0.0249026690093 | 0.366230866729 | 0.000360474896606 | 0.523600584955 | 0.0694315626504 | 0.00681579954273 | 0.00766964330704 | 0.0007829669797238069 |
