# Supplementary material for: Integrity of the Human Faecal Microbiota following Long-Term Sample Storage
Source: PLoS One. 2016 Oct 4;11(10):e0163666. doi: 10.1371/journal.pone.0163666 (PMC5049846; doi:10.1371/journal.pone.0163666)
Supplement: S2 Fig — [7] (blue symbols, n = 12) and a matched cohort of individuals from the American Gut Project (open symbols, n = 110). Results obtained using three different diversity metrics are shown (weighted UniFrac is the same as Fig 3 in the article). (PPTX) [file pone.0163666.s002.pptx]

## Slide 1
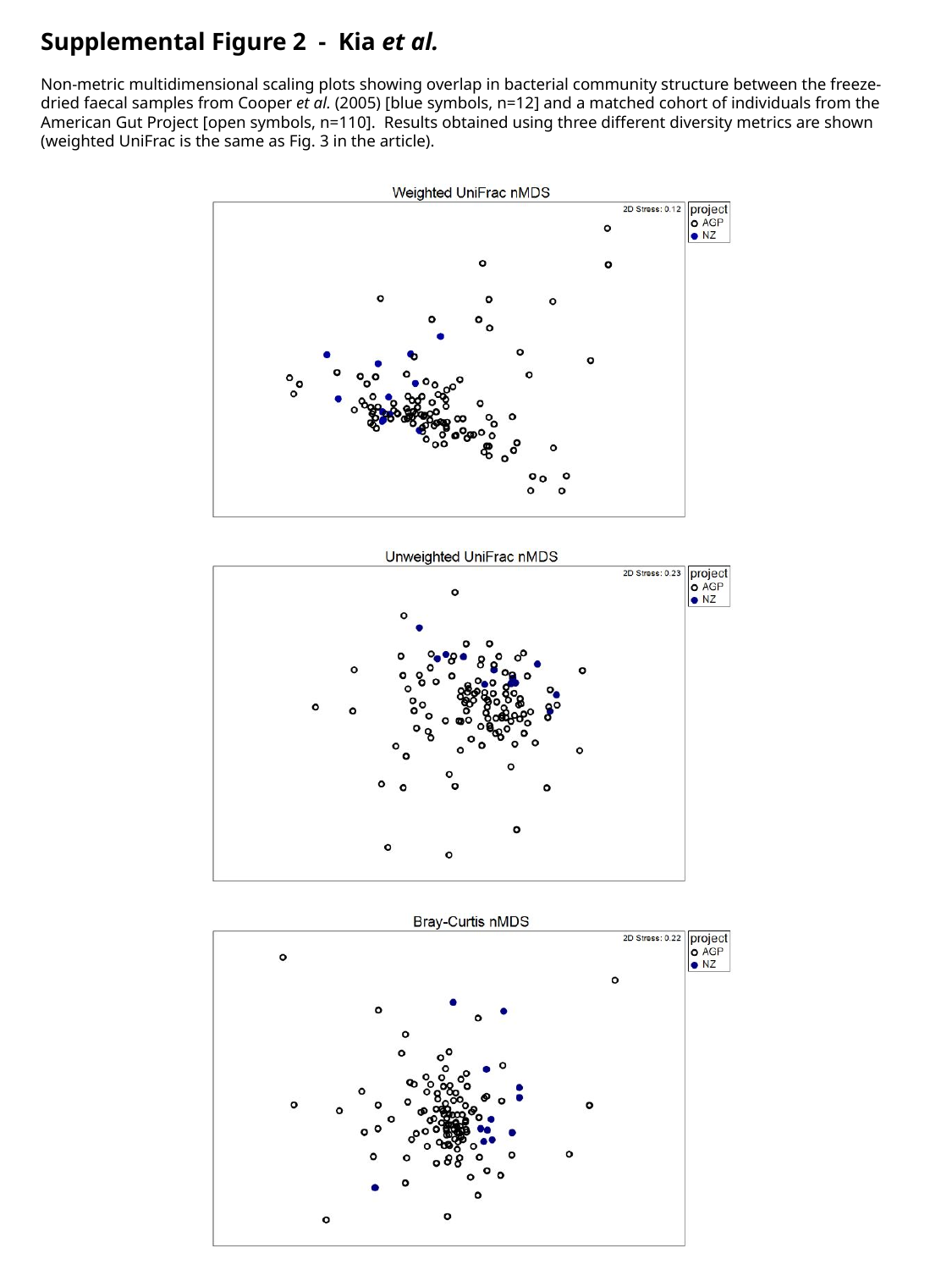

Supplemental Figure 2 - Kia et al.
Non-metric multidimensional scaling plots showing overlap in bacterial community structure between the freeze-dried faecal samples from Cooper et al. (2005) [blue symbols, n=12] and a matched cohort of individuals from the American Gut Project [open symbols, n=110]. Results obtained using three different diversity metrics are shown (weighted UniFrac is the same as Fig. 3 in the article).
